# Reducing the haystack to find the needle: improved protein identification after fast elimination of non-interpretable peptide MS/MS spectra and noise reduction

**DOI:** 10.1186/1471-2164-11-S1-S13

**Published:** 2010-02-10

**Authors:** Nedim Mujezinovic, Georg Schneider, Michael Wildpaner, Karl Mechtler, Frank Eisenhaber

**Affiliations:** 1 Sarajevo School of Science and Technology, Bistrik 7, Sarajevo 71000, Bosnia-Herzegovina; 2 Bioinformatics Institute (BII), A*STAR, Biopolis, 30 Biopolis Street, #07-01 Matrix Bldg., Singapore 138671; 3 Google Switzerland GmbH, Brandschenkestraße 110, 8002 Zuerich, Switzerland; 4 Research Institute of Molecular Pathology, Dr. Bohr-Gasse 7, A-1030 Vienna, Austria; 5 Department of Biological Sciences (DBS), National University of Singapore (NUS), 8 Medical Drive, Singapore 117597; 6 School of Computater Engineering (SCE), Nanyang Technological University (NTU), 50 Nanyang Drive, Singapore 637553

## Abstract

**Background:**

Tandem mass spectrometry (MS/MS) has become a standard method for identification of proteins extracted from biological samples but the huge number and the noise contamination of MS/MS spectra obstruct swift and reliable computer-aided interpretation. Typically, a minor fraction of the spectra per sample (most often, only a few %) and about 10% of the peaks per spectrum contribute to the final result if protein identification is not prevented by the noise at all.

**Results:**

Two fast preprocessing screens can substantially reduce the haystack of MS/MS data. (1) Simple sequence ladder rules remove spectra non-interpretable in peptide sequences. (2) Modified Fourier-transform-based criteria clear background in the remaining data. In average, only a remainder of 35% of the MS/MS spectra (each reduced in size by about one quarter) has to be handed over to the interpretation software for reliable protein identification essentially without loss of information, with a trend to improved sequence coverage and with proportional decrease of computer resource consumption.

**Conclusions:**

The search for sequence ladders in tandem MS/MS spectra with subsequent noise suppression is a promising strategy to reduce the number of MS/MS spectra from electro-spray instruments and to enhance the reliability of protein matches. Supplementary material and the software are available from an accompanying WWW-site with the URL http://mendel.bii.a-star.edu.sg/mass-spectrometry/MSCleaner-2.0/.

## Background

Liquid chromatography (LC) coupled with tandem mass spectrometry (MS/MS) is the method of choice for the identification of proteins extracted from biological samples. The standard procedure of post-MS/MS data processing involves computer-aided interpretation of the measured spectra with MASCOT [1], SEQUEST [2] or some other software for comparing theoretical spectra calculated for database sequences with the experimental ones. But modern instruments generate extremely large sets of MS/MS spectra (in the order of 10000 per sample), which are heavily contaminated with different types of background and noise. In addition to b-, y- and their derivative ions from peptides, spectra contain repeated shifted signals due to the natural isotope distribution (isotope clusters), multiply charged replicas, peaks from unknown fragmentation pathways, sample-specific or systematic chemical contaminations and random noise from the electronic detection system.

Thus, the spectra consist mostly of background; typically, only a few percent of the spectra recorded have signals from target protein fragments and just about 10% of the peaks in such a spectrum contribute to the peptide identification. Thus, computer resources in mass spectrometry departments all over the world are mostly spent on analyzing non-relevant data if the identification of the protein with significance is possible within the background at all. This strategy clashes with limitations in compute server capacity in proteomics laboratories and seriously limits the access of less generously equipped teams to the field.

With the broad availability of accurate MS/MS instruments with resolution in the order of tenths of a Dalton, automatic background removal procedures before interpretation software application became possible [3-5]. Various spectrum pre-processing rules, deconvolution of multiply charged peaks and deisotoping procedures have been described [6-15]. It should be noted that many spectra do not contain peaks from peptide fragmentations or are extremely noisy and, therefore, are non-interpretable into peptide sequences reliably. Thus, the exclusion of non-interpretable spectra is a valid strategy for reducing the computational load. For a well performing method, one would desire it to remove clearly more than half or three quarters of the experimental MS/MS spectra and, essentially, to keep all interpretable ones. At the same time, computation time for this task should be negligible or, at least, small compared to the processing time used by an interpretation program such as MSACOT that is saved by unselecting a large spectra subset.

Published approaches to this problem differ in the criterion for spectrum selection, either with empirically defined score functions or with a classifier generated by automated learning approaches [16-23]. Although many of these methods apply quite sophisticated criteria, they either are not efficient filters or suffer from a substantial fraction of unselected but nevertheless interpretable MS/MS spectra (e.g., loss of ~10% of the interpretable spectra for removing ~75% of the total number spectra in Figures 2 and 3 of Bern *et al. *[18]). Thus, substantial computational load reduction is traded in for the risk not to find the desired peptide hit. Consequently, none of the published techniques has routinely entered the laboratories so far.

In the attempt to develop an alternative methodical approach, we propose to return to ideas from the beginning of mass spectrometry of proteins. Originally, interpretation of an MS/MS spectrum meant experts trying to manually find sequence ladders (i.e., sets of peaks with amino acid mass spacing between them) among the high-intensity peaks. The concept of searching mainly among the higher intensity peaks is still reminiscent in the formulas for evaluating the significance of a peptide hit as used in MASCOT [1]. Indeed, a peptide the theoretical fragmentation spectrum of which matches exclusively low intensity peaks cannot serve as convincing explanation of the experimental data.

In this work, we explore the idea that at least some short oligopeptide segment of a significant peptide hit should be fully matched by the higher intensity peaks in the spectrum. In an efficient implementation, the computational costs are low if one tries just to check whether small peptide ladders of predefined length do occur in a MS/MS spectrum at all among the top fraction of most intense peaks. The identity of the oligopepetide is not important in this context; it is rather questioned whether such an amino acid chain theoretically exists at all. It is reasonable to suggest that the spectrum is probably not interpretable into a peptide sequence with statistical significance if not even a short oligopeptide sequence is matched by this criterion.

After this unselecting procedure, the remaining spectra still contain considerable background in the typical case. In a previous publication [24], we developed an approach based on techniques from electrical signal processing. Periodical band-reject and high-frequency filters as well as correlation analyses with etalons of multiply charged clusters can successfully be used for background suppression. In this work, we describe a workflow involving sequence ladder and improved signal processing criteria on a large MS/MS dataset exemplified in the MS Cleaner version 2.0 that efficiently reduces the number and the size of spectra and, subsequently, dramatically shrinks the computing time used by the interpretation software. To emphasize, the approach described in this work is thought to increase the efficiency of protein identification. It is not considered to process MS/MS data that is intended to be screened for protein posttranslational modifications.

## Methods

### Mass spectrometry

Commercially acquired proteins (α-amylase, amyloglucosidase, apo-transferrin, β-galactidase, carbonic anhydrase, catalase, phosphorylase B, glutamic dehydrogenase, glutathione transferase, immunoglobulin γ, lactic dehydrogenase, lactoperoxidase, myoglobin) were used, each in two independent preparations (each with a concentration of 100 fmol). For chromatography, a UltiMate Plus Nano-LC system. LC-Packings - A Dionex Co was used. Chromatographic mobile phases were: loading mobile phase 0.1% TFA in water, separation mobile phase A 5% acetonitrile in 0.1% aqueous formic acid and mobile phase B 80% acetonitrile, 20% water with 0.08% formic acid. The sample was loaded for 10 min onto a reversed phase trap column (PepMap C18, 300 μm ID × 5 mm length, 5 μm particle size, 100 Å pore size, LC Packings - A Dionex Co., not online with the separation column) at a flow rate of 20 μl/min and washed free of ion pairing agents and other impurities.

The gradient for separation of analytes starts at 10 min when the trap column is switched online with the separation column (PepMapC18, 75 μm ID × 15 cm length, 3μm particle size, 100 Å pore size) at 0.275 μl/min. The gradient used starts at 100% mobile phase A and changes to 50% mobile phase B from 10 minutes (trap column and separation column online) to 40 minutes. Additional wash step of 90% mobile phase B is incorporated in order to clean the separation column and elute hydrophobic analytes. After the separation, the trap column is switched offline and equilibrated with loading mobile phase. The analytical nano column is equilibrated with separation mobile phase A. The mass spectrometric data are only recorded for the time both columns are online.

The mass spectra were recorded with a Thermo Finnigan LTQ (positive nano-ESI mode, ionizing spray voltage: 1.5 kV, enhanced mass-spec full-scan range: 220 - 2000 amu). The much smaller datasets for bovine serum albumin (BSA), yeast alcohol dehydrogenase (ADH) and human transferrin (TRF) recorded with a 3D IT mass spectrometer (model DecaXP Thermo Finnigan) were reused from our previous work [24].

### File processing and MS/MS data analysis

The MS/MS output was converted into mgf-files (MASCOT generic format). Each dataset was then separately processed using the MS Cleaner program (with default internal parameters), generating two new mgf-files with cleaned and bad (non-interpretable) spectra respectively. The MASCOT search parameters were the same in all runs (enzyme: trypsin; fixed modifications: carbamidomethyl (at cysteines) for BSA, ADH and TRF, carboxymethyl (at cysteiness) for other proteins; variable modifications: oxidation (at methionines); peptide charges: 1+, 2+ and 3+; mass values: monoisotopic; protein mass: unrestricted; peptide mass tolerance: ± 2 Da; fragment mass tolerance: ± 0.8 Da; max. missed cleavages: 1). The MASCOT search results output html-file was formatted with standard scoring, a significance threshold of p < 0.05, and an ion score cut-off for each peptide of 30. The non-redundant protein database (NCBI) was used (both for the local PC MASCOT installation and for the MASCOT Linux cluster).

In this work, we compare the MASCOT interpretation results of non-pre-processed tandem MS datasets with those obtained in a two-step preprocessing. First, each spectrum (.dta-file) is analyzed with the sequence ladder algorithm. Only those spectra that pass this test, are then processed with the background removal routines described in our previous publication [24].

### The sequence ladder algorithm

For this algorithm, two parameters are critical - the values ***n ***(in amino acid residues), the minimal length of the sequence ladder, and ***s ***(in per cent), the fraction of peaks from the spectrum that is considered of high intensity. The number ***n ***can theoretically be just one (i.e., we would require just two high intensity peaks that are spaced by the mass difference corresponding to the mass of one of the amino acids); yet, larger values of ***n ***(for example, between two and six residues) represent stricter requirements to the sequence ladder. The other parameter ***s ***restricts the search space. For this purpose, the peaks in the spectrum considered (i.e., in one .dta-file) are sorted by intensity into a list with descending order. Only the first part of this list (the fraction ***s ***of the total set) is used for searching sequence ladders. The condition of ***s ***= 100% implies that all peaks are included; yet, considerably smaller values of ***s ***are desirable since they would help unselecting more non-interpretable spectra. Once the set of high-intensity peaks is defined, their pair-wise mass differences are compared in a systematic enumeration with the masses of amino acids residues (to select pairs of peaks separated by the mass of any of the amino acids within a user-defined accuracy) and it is tested whether a subset of peaks forms a sequence ladder of the required minimal length. If at least one such ladder is found, the search is stopped and the procedure is restarted with the next tandem MS spectrum in the dataset.

### Modifications of the noise detection algorithm

If a spectrum has passed the sequence ladder test, it is handed over to a series of routines for noise and background detection. The procedures for removing multiply charged peak clusters with the etalon method and for the suppression of high-frequency noise with a low-pass filter after Fourier transformation have been described in a previous publication [24] in detail and have been applied without changes here.

The algorithm for the removal of latent periodic background (including deisotoping) received another option with respect to the determination of the base frequency of the noise. We observed that the determination of the base frequency f_*B *_in the first power spectrum (see sections 3.3 and 3.5 in ref. [24]) is, in rare cases, not always as unambiguous as in Figure 2A of ref. [24] since several almost equally intense peaks may appear in the second-level Fourier transform. Wrong base frequency f_*B *_detection leads to wrong multi-band rejection filter creation and a few interpretable spectra can be lost after applying this technique. This ambiguity can be avoided by not choosing the frequency of the most intense peak in the second-level Fourier transform. Rather, we propose to iterate through all possible base frequencies detected in this spectrum. For each of these frequencies, theoretical maxima and minima expected in first level Fourier transform are calculated. Best matching between the theoretical and experimental maxima and minima (see Figure 3 in ref. [24]) confirms the right base frequency. We call this method "soft recognition" of latent periodic noise which should be applied if minor improvements in sequence coverage (in rare cases, a single additional peptide) are more important than data size reduction; yet, it leads to an increment of about 10% of the computation time compared with the previous method [24].

### Standalone implementation and cluster version

We created two implementations for MS Cleaner 2.0. A single-machine Windows version was used for most of the computations in this article and it is available for free download at the associated WWW site. A Unix-Port of the MS Cleaner 2.0 software is deployed in a clustered environment in order to guarantee scalability. The spectrum file is partitioned into workpackages, which are then handed over to a batch queuing system for scheduling on available nodes. Each node processes the spectra in its workpackage and transfers the results back to the controlling application where they are post-processed into the final good/bad spectra output. This version is the engine behind the MS Cleaner 2.0 WWW server.

### WWW Supplement

At the WWW-site http://mendel.bii.a-star.edu.sg/mass-spectrometry/MSCleaner-2.0/, supplementary resources are available: all experimental mass-spectrometry data used in this work, the processed spectra, the user manual, default parameter datasets and a free downloadable Windows version of the program MSCleaner 2.0 as well as free access to a MSCleaner 2.0 WWW server accessing a local Linux cluster. Other implementations can be obtained on request.

## Results and discussion

For the initial determination of optimal parameter ranges (sequence ladder length ***n ***and peak intensity threshold ***s***), we used the datasets for bovine serum albumin (BSA), yeast alcohol dehydrogenase (ADH) and human transferrin (TRF) from our previous work [24] since they are quite small (less than 3000 .dta-files per set). We checked the influence of the preprocessing procedures on the spectrum interpretation with the MASCOT tool. A systematic analysis was performed; sequence ladder length was tested with values ***n ***between 2 and 6 and the high-intensity threshold ***s ***was varied from 5% to 35% (the sequence ladder was searched for only among the 5%, 10%, 15%, ..., or 35% of most intense peaks). The goal is to have as many unselected "bad" spectra as possible (the savings in computing time are about proportional to the fraction of spectra that is not handed over to the spectrum interpretation program) without losses of (i) MASCOT score, (ii) spectra giving peptide matches and (iii) sequence coverage.

Due to the space limitation, only the results of a parameter subset are presented (Table 1). As expected, the number of detected bad spectra increases with growing sequence ladder length ***n ***and decreasing intensity threshold ***s***. We observe that the MASCOT score of the non-preprocessed data (586 for BSA, 224 for ADH and 588 for TRF; see rows with ***n ***= 0 and ***s ***= 0%) is considerably smaller than that of the cleaned datasets (often, by a factor of 2-5) regardless of the severity of data pre-processing. Thus, the reliability of the top protein hit in the database searches greatly increases by the background reduction, both by discarding bad spectra and by removing noise from spectra that can be interpreted in peptides. This alone is an interesting result.

**Table 1:** Influence of background removal on the recovery of BSA, ADH and TRF in MS/MS spectra of 100 fmol test samples. The original number of MS/MS spectra for the BSA (bovine serum albumine), ADH (yeast alcoholdehydrogenase) and TRF (human transferring) datasets (recorded on a DecaXP machine) are 2679, 2325 and 2608 respectively. The intensity threshold *s *(column 3) describes the search of the sequence ladder (length *n *in column 2) within the 15%, 20%, 25% or 30% top peaks (100% - all peaks are considered). The following three columns show the MS Cleaner output - number of spectra with background removal, number of unselected spectra and the MS Cleaner CPU time on a single-processor Windows XP computer (Pentium IV 2.4 GHz; to get exact measurements of computation time, we did not use the cluster version). The remaining four columns present the MASCOT output - the CPU time on the same machine, the protein score, the number of spectra matching peptides in a MASCOT search and the final sequence coverage. For each dataset, the first line shows the results for the case when MS Cleaner is not used for pre-processing and the MS/MS data is immediately interpreted by MASCOT.

protein	sequence ladder length *n*	intensity threshold *s *[%]	cleaned spectra	bad spectra	MS Cleaner time [min]	MASCOT time [min]	MASCOT score	queries matched	sequence coverage
BSA	0	100	-	-	-	61	586	89	55

	3	100	1664	1015	3.92	44	720	91	57
	3	15	390	2289	1.21	17	1991	84	52
	3	20	490	2189	1.40	21	2108	87	57
	3	25	601	2078	1.61	26	2114	89	57
	3	30	688	1991	1.75	29	2114	90	57

	4	100	940	1739	3.80	36	2108	91	57
	4	15	260	2419	0.91	12	1875	78	47
	4	20	321	2358	1.06	14	1911	80	47
	4	25	380	2299	1.25	18	2114	86	57
	4	30	441	2238	1.30	19	2114	89	57

	5	100	593	2086	3.82	26	2108	91	57
	5	15	174	2505	0.60	9	1579	60	41
	5	20	232	2447	0.85	11	1809	72	44
	5	25	281	2398	1.00	13	1963	81	49
	5	30	313	2366	0.85	14	2058	86	54

ADH	0	100	-	-	-	64	242	39	39

	3	100	1446	879	4.15	45	327	34	39
	3	15	269	2056	0.88	12	673	29	35
	3	20	347	1978	1.10	13	696	31	37
	3	25	440	1885	1.33	17	697	32	37
	3	30	697	1628	1.53	20	697	33	37

	4	100	902	1423	4.15	35	733	34	39
	4	15	173	2152	0.58	7	562	26	28
	4	20	216	2109	0.71	9	673	30	35
	4	25	271	2054	0.90	12	607	28	33
	4	30	325	2000	1.05	13	697	32	37

	5	100	594	1731	4.20	23	712	33	39
	5	15	94	2231	0.35	5	311	15	21
	5	20	125	2200	0.46	6	366	17	25
	5	25	145	2180	0.53	7	434	19	26
	5	30	186	2139	0.66	9	589	24	31

TRF	0	100	-	-	-	52	588	86	47

	3	100	1587	1021	3.57	42	768	87	49
	3	15	373	2235	1.00	17	1988	86	49
	3	20	485	2123	1.23	20	1988	86	49
	3	25	568	2040	1.36	24	1998	87	49
	3	30	639	1969	0.78	27	1998	87	49

	4	100	864	1744	3.62	34	1973	87	49
	4	15	231	2377	0.70	11	1987	81	49
	4	20	298	2310	0.86	13	1988	84	49
	4	25	360	2248	1.00	16	1988	85	49
	4	30	414	2194	1.12	19	1998	87	49

	5	100	540	2068	3.63	23	1973	87	49
	5	15	164	2444	0.55	9	1785	68	45
	5	20	194	2414	0.61	10	1890	74	47
	5	25	245	2363	0.75	12	1957	80	48
	5	30	286	2322	0.86	14	1968	84	48

The sequence coverage is more sensitive to the pre-processing parameters. For a sequence ladder length of ***n ***= 5 residues, we see a trend that sequence coverage is slightly decreased with respect to that of unprocessed data (41-54% instead of 55% for BSA, 21-31% instead of 39% for ADH, 45-48% instead of 47% for TRF). Sequence coverage is about the same or even slightly higher as for non-preprocessed data for sequence ladder lengths ***n ***= 3 and ***n ***= 4 and intensity thresholds ***s ***at and above 20%. With regard to the number of spectra that lead to a significant peptide match in the MASCOT search, the settings ***n ***= 3, ***s ***= 20%; ***n ***= 3, ***s ***= 25%; ***n ***= 4, ***s ***= 20% and ***n ***= 4, ***s ***= 25% are close to reproduce the result achieved with the unprocessed data for the BSA and TRF cases. Surprisingly, the number of peptide matches is slightly higher for ***s ***= 100% (all peaks are included in the sequence ladder search) than for the datasets without preprocessing. Thus, the number of falsely rejected spectra by the sequence-ladder algorithm is essentially zero in these two cases. For ADH, the number of spectra matching peptides is always somewhat lower if the tandem MS/MS data is pre-processed, although MASCOT score and sequence coverage do not suffer from choices of ***n ***= 3, or ***n ***= 4 and the higher values of ***s***.

To detect a considerable fraction of the bad spectra and to reduce the time for interpretation by MASCOT, these results support the selection of a sequence ladder length equal to ***n ***= 4 and an intensity threshold of ***s ***= 20%. If the sequence coverage is more important than computational time savings, softer parameters can be chosen, for example with an intensity threshold of ***s ***= 25%. With these parameters, it is possible to eliminate more than 80% of all spectra in the datasets BSA, ADH and TRF by declaring them non-interpretable in oligopeptides (see Table 1). Minor sequence coverage loss, if at all observed, does not affect the interpretation result. Yet, the total computing time required for interpretation narrows up to only 20% of the original value. The computing time consumption for MS Cleaner alone in such a setting is ~2% of MASCOT time for non-preprocessed data (see Table 1); i.e., it is essentially negligible.

For further analysis of the algorithm's performance, large MS/MS datasets are necessary that are recorded from samples with known protein composition. For this purpose, we used solutions of commercially available proteins at 100 fmol concentration. The behavior of the MS Cleaner algorithms was tested over this large dataset of about 270000 spectra from 26 samples of 13 proteins (Table 2) generated by an LTQ device. We used sequence ladder length ***n ***= 4 with intensity thresholds ***s ***= 20% and ***s ***= 25% and contrasted the results both (i) with the MASCOT-based interpretation of non-preprocessed data and (ii) with sequence ladder 4 and the inclusion of all peaks (***s ***= 100% threshold). We find that, as a rule, preprocessing reproduces or slightly improves the sequence coverage relative to the non-preprocessed data (100-110% for threshold ***s ***= 100% (columns A4 and A7), 100-108% for thresholds ***s ***= 20% and ***s ***= 25% (columns A4, A11 and A15)). Thus, the number of falsely rejected spectra by the sequence-ladder algorithm is essentially zero in these examples. This clear trend says that the preprocessing algorithm proposed here performs even better if it is supplied with more accurate data from the LTQ instrument as compared with those from the DecaXP. There is a trend for increased MASCOT scores (103-140% for threshold 100% (columns A3 and A6), 98-140% for ***s ***= 20% (A3 and A10) and 103-140% for ***s ***= 25% (A3 and A14) with an average of 110% regardless of threshold. The reduction of the dataset by unselecting spectra is significant (on average, 11% for threshold 100% (column A5), 63% for threshold ***s ***= 20% (column A8) and 53% for threshold ***s ***= 25% (column A13)). This means that the interpretation time with MASCOT reduces in a similar proportion.

**Table 2:** Performance of the MSCleaner version 2.0 over a large test set.

A1	A2	A3	A4	A5	A6	A7	A8	A9	A10	A11	A12	A13	A14	A15	A16
**alphaAmyl_col1**	10108	633	24	11.30	667	24	31.65	60.07	667	24	15.13	51.09	667	24	18.07
**alphaAmyl_col2**	10184	698	35	9.82	780	35	34.20	50.22	780	35	19.05	20.25	780	35	22.76
**AmylGlu_col1**	10030	736	28	13.26	761	28	28.40	79.24	761	28	8.66	73.58	761	28	10.63
**AmylGlu_col2**	9870	801	36	13.31	860	37	29.50	72.62	860	37	11.70	63.95	860	37	14.29
**apo_col1**	10032	2606	63	11.72	2814	63	30.76	63.10	2814	63	13.93	54.49	2814	63	16.78
**apo_col2**	10090	2571	60	12.13	2761	60	32.95	53.12	2761	60	17.53	44.32	2761	60	21.03
**betaGal_col1**	10324	1459	56	7.17	1567	57	34.98	48.06	1567	57	22.05	40.53	1567	57	24.60
**betaGal_col2**	10368	1309	51	8.12	1508	56	36.71	42.90	1454	55	24.76	33.10	1454	55	28.61
**CarAnly_col1**	9946	586	49	12.35	616	49	26.35	90.31	573	49	3.65	84.94	607	49	5.48
**CarAnly_col2**	9534	582	52	13.40	616	52	26.27	86.07	616	52	5.08	78.44	616	52	7.66
**Cat_col1**	10098	1798	61	11.13	1886	61	30.88	67.26	1879	61	13.13	57.89	1879	61	16.50
**Cat_col2**	10034	1567	65	11.78	1693	65	31.90	59.50	1693	65	15.91	48.55	1693	65	19.56
**phosB_col1**	10118	2780	59	10.30	3079	61	35.13	63.49	3014	60	14.26	54.46	3047	61	17.25
**phosB_col2**	10096	2655	61	10.52	3116	65	32.58	53.96	3084	65	17.58	44.31	3116	65	21.16
**GluDey_col1**	10006	892	36	11.29	986	36	27.30	79.55	986	36	7.75	73.42	986	36	9.71
**GluDey_col2**	9886	850	34	11.81	962	34	28.73	72.51	962	34	10.13	62.25	962	34	13.51
**GluTra_col1**	10022	351	25	10.36	389	25	28.61	71.64	348	25	10.25	62.78	389	25	14.30
**GluTra_col2**	10156	341	33	9.18	384	33	31.31	61.15	384	33	14.25	49.59	384	33	28.11
**Immo_col1**	10330	506	35	9.27	565	35	36.20	42.30	565	35	24.95	34.44	565	35	27.66
**Immo_col2**	10334	356	66	8.61	500	66	38.05	37.06	500	66	27.31	28.47	500	66	30.31
**LacDe_col1**	10286	1549	58	10.36	1694	58	35.36	53.20	1694	58	20.03	44.86	1694	58	23.15
**LacDe_col2**	10250	1346	54	9.07	1483	54	36.48	40.16	1483	54	25.60	31.67	1483	54	28.31
**LactoPee_col1**	10242	1613	45	13.16	1764	45	34.78	62.12	1756	45	15.91	52.37	1764	45	19.53
**LactoPee_col2**	10402	1679	43	9.09	1890	44	35.18	51.70	1890	44	20.31	41.76	1890	44	23.85
**Myo_col1**	9958	561	66	11.67	594	66	27.26	85.42	594	66	5.46	79.25	594	66	7.45
**Myo_col2**	9744	530	66	12.15	584	66	28.01	80.83	584	66	6.95	70.92	584	66	10.35

A1 name of test set (.mgf file; see Methods),

A2 total number of spectra (.dta files),

A3 MASCOT score of top protein hit with the original .mgf file

(without application of MS Cleaner),

A4 sequence coverage (in %) without application of MS Cleaner,

A5 fraction of non-interpretable "bad" spectra found with sequence ladder

length ***n ***= 4 among all peaks (intensity threshold ***s ***= 100%)

A6 MASCOT score of the top protein hit for this search,

A7 sequence coverage (in % of the whole protein length) for this search,

A8 MS Cleaner processing time (in min) on a PC with a single Pentium IV (to

achieve exact time consumption values, we did not use the cluster version and

stopped the "soft frequency recognition option")

A9 fraction of non-interpretable "bad" spectra found with sequence ladder

length ***n ***= 4 among the ***s ***= 20% most intense peaks

A10 MASCOT score of the top protein hit for this search,

A11 sequence coverage (in % of the whole protein length) for this search,

A12 MS Cleaner processing time (in min),

A13 fraction of non-interpretable "bad" spectra found with sequence ladder

length ***n ***= 4 among the ***s ***= 25% most intense peaks (in % of A2; i.e.,

of all spectra)

A14 MASCOT score of the top protein hit for this search,

A15 sequence coverage (in % of the whole protein length) for this MASCOT

search,

A16 MS Cleaner processing time on the same machine as described in the legend of

Table 1 (in min).

The sequence ladder criterion (minimal ladder length 4 with varying peak intensity thresholds) and the noise suppression algorithms of MS Cleaner 2.0 have been applied over a large set of tandem MS results. For each of the test proteins, two independent sample preparations and dataset recordings (marked with appendices _col1 and _col2 in the dataset name) were carried out: α-amylase, amylogucosidase, apo-transferrin, β-galactidase, carbonic anhydrase, catalase, phosphorylase B, glutamic dehydrogenase, glutathione transferase, immunoglobulin γ, lactic dehydrogenase, lactoperoxidase, myoglobin). For these datasets, the MASCOT interpretation was carried out on a cluster in parallel with other jobs; therefore, no computation time is provided.

To summarize, the results support that testing spectra for interpretability in oligopeptides is a useful criterion for dataset reduction in protein mass spectrometry if a sequence ladder of a tetrapeptide segment is searched for among the 20% (or 25%) most intense peaks. This preprocessing is accompanied by an increase in MASCOT score and more significant top protein hits and it does not significantly affect sequence coverage. Running MS Cleaner 2.0 as a standard preprocessing step in peptide tandem MS data analysis for protein identification is recommended.

The idea of using short series of sequence ions (peptide sequence tags) as a specific identifier that speeds up searches for matches between spectra and sequences in databases (either by searching the database with the tag or by creating sequence tag database filters in order to reduce the size of a database via a preprocessing step) is extensively explored in the literature [25-27]. It is interesting to see that this simple idea applied to the problem of recognizing spectra non-interpretable in oligopeptides greatly reduces the complexity of analyzing protein mass spectrometry data.

## List of abbreviations used

CID: collision-induced dissociation; Da: Dalton; ESI: Electrospray ionization; LC-MS/MS: liquid chromatography coupled with tandem mass spectrometry; MS: mass spectrometry; MS/MS: tandem mass spectrometry; PS: power spectrum.

## Competing interests

The authors declare that they have no competing interests.

## Authors' contributions

NM programmed the single-processor prototype of MSCleaner and carried out all computational experiments. NM and FE together produced the WWW site associated with this publication. GS and MW were instrumental for creating the multi-processor version and the WWW server. KM provided the wet lab part of this work and participated in the discussion of the results. FE proposed the scientific task, guided the work and wrote the article.
